# Ethnic differences in carotid bulb geometry between Asian and White populations

**DOI:** 10.3389/fneur.2026.1805941

**Published:** 2026-04-21

**Authors:** Hossam Abdelmageed, Lamia Mbarek, Xuewei Xie, Yongjun Wang, Hui Li, Yang Liu, Huaguang Zheng, Robert Fleischmann, Jens P. Dreier, José Manuel Valdueza

**Affiliations:** 1 Neurology Department, University of Greifswald, Greifswald, Germany; 2 China National Clinical Research Center for Neurological Diseases, Beijing, China; 3 Department of Neurology, Beijing Tiantan Hospital, Capital Medical University, Beijing, China; 4 Department of Clinical Epidemiology and Clinical Trial, Capital Medical University, Beijing, China; 5 Health Management Center, Beijing Tiantan Hospital, Capital Medical University, Beijing, China; 6 Center for Stroke Research Berlin, Charité–Universitätsmedizin Berlin, Corporate Member of Freie Universität Berlin, Humboldt-Universität zu Berlin, Berlin Institute of Health, Berlin, Germany; 7 Experimental Neurology, Charité–Universitätsmedizin Berlin, Corporate Member of Freie Universität Berlin, Humboldt-Universität zu Berlin, Berlin Institute of Health, Berlin, Germany; 8 Neurological Center, Segeberger Kliniken, Bad Segeberg, Germany

**Keywords:** carotid bulb, stenosis, carotid ultrasound, race-ethnic, atherosclerosis, white-Asian, cardiovascular risk factors, stroke

## Abstract

**Background:**

Carotid bifurcation geometry influences local hemodynamics and may contribute to plaque formation and carotid disease. Interethnic differences in carotid bulb geometry remain incompletely characterized. However, Asian populations are known to have less atherosclerosis burden in the extracranial carotid arteries compared to White populations.

**Methods:**

In a cross-sectional study (2022–2025), 200 adults without relevant carotid stenosis were enrolled consecutively (100 White participants in Germany; 100 Asian participants in China). Carotid geometry was assessed bilaterally using predefined diameter ratios; Carotid bulb (CB)/Internal carotid artery (ICA) diameter was the prespecified primary outcome. Linear mixed-effects models accounted for within-subject correlation; adjusted models included age, sex, BMI, diabetes, hypertension, dyslipidemia, smoking, and atrial fibrillation.

**Results:**

In unadjusted models, significant ethnic differences were observed across all primary geometry ratios. CB/ICA was lower in Asian versus White participants (EMM 1.57 vs. 1.68; *p* < 0.001). After adjustment, the CB/ICA difference remained robust (Asian minus White −0.09; 95% CI -0.14 to −0.04; *p* < 0.001). Additional adjusted differences were observed for CB/CCA, ICA/CCA, ECA/ICA, and outflow/inflow, whereas no significant difference persisted for ECA/CCA. Analyses of absolute diameters showed no significant interethnic difference in ICA diameter, while carotid bulb diameter was smaller in Asian participants.

**Conclusion:**

Carotid bifurcation geometry differs between Asian and White adults independent of vascular risk factors. These anatomical differences may have implications for carotid hemodynamics and plaque formation across populations.

## Introduction

Ischemic stroke remains a major global health problem and is among the leading causes of death and long-term disability worldwide (1). Among the various ischemic stroke subtypes, large artery atherosclerosis (LAA) accounts for a substantial proportion of cases and remains a major contributor to global stroke burden. Atherosclerotic disease of cerebral arteries, affecting both extracranial and intracranial vessels, is one of the most common underlying mechanisms. Importantly, the anatomical distribution of atherosclerosis differs between populations. Intracranial atherosclerotic disease (ICAD) is observed more frequently in Asian populations (2, 3) whereas extracranial carotid stenosis predominates in White populations (4). While these differences have often been attributed to variations in lifestyle and traditional cardiovascular risk factors, such explanations do not fully account for the consistent ethnicity divergence in disease patterns.

In recent years, increasing attention has been directed toward the role of vascular anatomy in atherosclerosis development (5). In particular, the carotid bifurcation is a key site where vessel geometry significantly influences local blood flow. Simulated-based models and in-vivo imaging studies have demonstrated that geometric features of carotid bifurcation critically determine wall shear stress (WSS) distribution patterns along the vessel wall. Regions exposed to low or oscillatory shear stress exhibit increased susceptibility to endothelial dysfunction and early atherogenesis (6), whereas areas subjected to uniform, high shear stress maintain relative protection against plaque formation. These hemodynamic conditions, which arise from the inherent vessel geometry, can manifest long before the development of significant luminal stenosis, highlighting the crucial role of biomechanical factors in the initiation and localization of atherosclerotic disease (6).

The carotid bulb (CB) represents a physiological dilation where flow deceleration and recirculation commonly occur. A larger bulb diameter has been associated with lower local shear forces (7), thereby expanding regions of low and oscillatory WSS that favor atherogenesis. In addition to bulb size, the distribution of blood flow between the internal and external carotid arteries further modulates shear stress within the bifurcation. A reduced contribution of flow into the internal carotid artery (ICA) may promote flow stagnation within the CB, whereas a higher internal carotid flow tends to limit low-shear regions. Together, CB geometry and flow distribution define the local hemodynamic environment at this critical vascular site (8–10).

Despite the recognized importance of carotid bifurcation geometry, ethnic differences in CB morphology have received little systematic investigation. It remains unclear whether the relative dimensions of the CB and its relationship to downstream vessels differ between Asian and White Populations. Such differences, if present, may represent a structural factor contributing to population-specific patterns of extracranial atherosclerotic disease beyond traditional vascular risk factors.

The present study, therefore aimed to examine ethnic differences in CB morphology in White and Asian individuals using extracranial ultrasound. We hypothesized that both the relative diameter of the CB and the outflow-inflow relationship at the carotid bifurcation differ between these populations. By characterizing these geometric features, this study seeks to improve our understanding of population-specific vascular anatomy and its potential influence on local hemodynamic conditions, which may, in turn, be relevant to ethnic differences in atherosclerosis and, subsequently, ischemic stroke.

## Methods

### Study design and participants

This cross-sectional observational study included two ethnically distinct adult populations: White participants recruited in Germany and Asian participants recruited in China between 2022 and 2025. A total of 200 participants were enrolled consecutively, comprising 100 individuals from each population group. Participants were recruited from inpatients and outpatients undergoing routine clinical evaluation, as well as from healthy volunteers among hospital staff. Inclusion criteria were age ≥18 years, ability to provide written informed consent, absence of clinically relevant carotid artery stenosis, and predefined ethnicity at each study site (White participants in the German cohort and Asian participants in the Chinese cohort). Ethnicity (Asian or White) was recorded by self-report at the time of recruitment.

Clinical data collected at study entry included age and sex. Body weight and height were measured, and body mass index (BMI) was calculated as weight in kilograms divided by height in meters squared (kg/m^2^). Information on cerebrovascular risk factors, including hypertension, diabetes mellitus, dyslipidemia, atrial fibrillation, and smoking status, was obtained from medical history and review of available medical records. Hypertension, diabetes mellitus, and hypercholesterolemia were defined based on documented medical history and/or current use of disease-specific medication. Cardiovascular risk factors were assessed using medical records. In healthy volunteers, these variables were obtained through structured history-taking, including confirmation of relevant medication use. All participants were assessed using identical inclusion criteria and underwent the same standardized ultrasound examination protocol, irrespective of recruitment source or study center.

The study was conducted in accordance with the Declaration of Helsinki and was approved by the local ethics committees of the participating centers (Greifswald, Germany: approval number BB 031/22; Beijing, China: approval number KY2025-198-01). All participants provided written informed consent prior to inclusion.

### Ultrasound acquisition

Extracranial carotid ultrasound examinations were performed by two trained investigators, one at each study center. The White cohort was examined at the University of Greifswald, Germany, and the Asian cohort at Beijing Tiantan Hospital, Capital Medical University, Beijing, China. Overall examination quality and adherence to the predefined imaging protocol were supervised and reviewed by the last author.

At both centers, high-resolution linear transducers with a nominal center frequency of approximately 7.5 MHz were used. In Germany, examinations were performed using a Vivid™ E95 system (GE Vingmed Ultrasound, Horten, Norway) with a 9L linear array transducer (3–10 MHz). In China, examinations were conducted using an Aplio 500 ultrasound system (Canon Medical Systems, Otawara, Japan) with an 11 L4 linear array transducer (4–11 MHz).

### Image acquisition and measurements

B-mode longitudinal images were used to measure luminal diameters of the distal common carotid artery (CCA), CB, ICA, and external carotid artery (ECA). Measurements were obtained using an electronic caliper with a precision of 0.1 mm. The outflow/inflow ratio was defined as the ratio of the summed cross-sectional areas of the internal and external carotid arteries to that of the common carotid artery.

**Figure 1:**
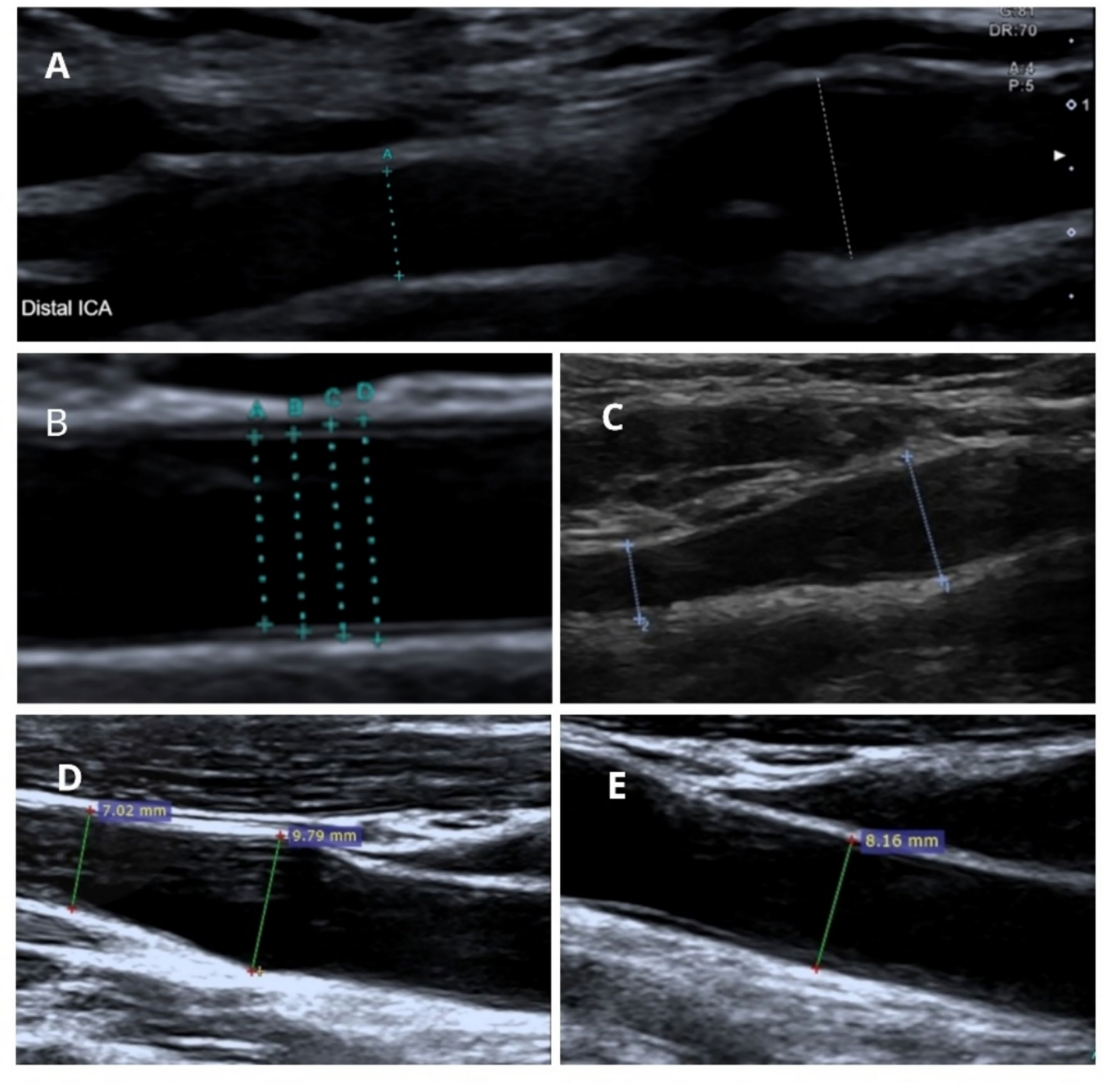
Ultrasound acquisition and measurement of carotid bifurcation geometry. Representative ultrasound images illustrating the standardized acquisition protocol of the carotid bifurcation. **(A,C,D)** Longitudinal ultrasound views demonstrate optimal visualization of the carotid bifurcation, with the common carotid artery (CCA), carotid bulb (CB), and internal carotid artery (ICA) visualized as a continuous structure. **(B)** Illustrates the media-to-media measurement principle. Four example measurement lines **(A–D)** are shown to demonstrate correct and incorrect positioning; only measurement **D** represents the correct technique used for analysis. Measurements were performed at end diastole in a plaque-free segment with parallel vessel walls. **(E)** Measurement of the common carotid artery diameter in the distal CCA segment proximal to the bifurcation.

**Figure 2:**
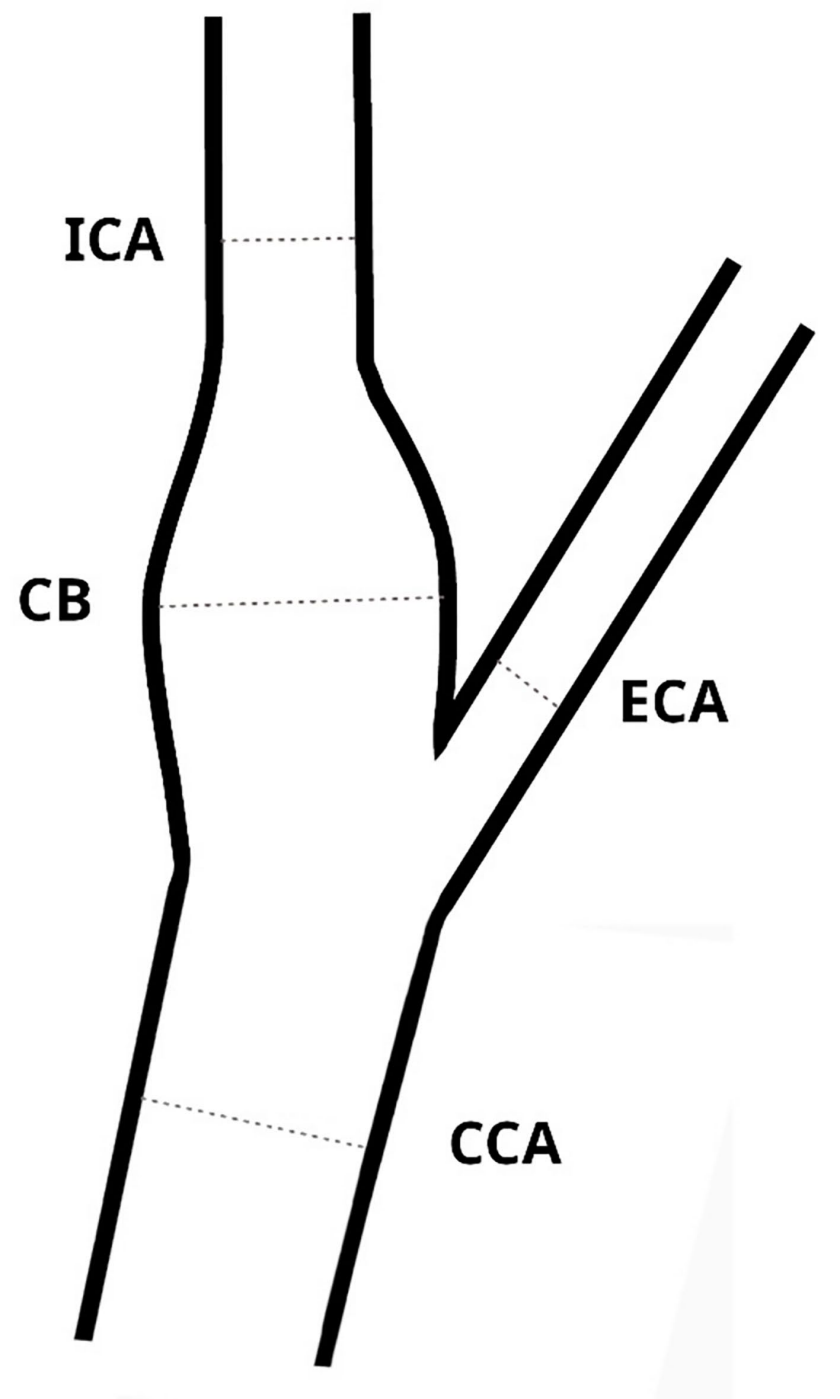
Schematic illustration of carotid bifurcation geometry and measurement locations. Diameters of the common carotid artery (CCA), carotid bulb (CB), internal carotid artery (ICA), and external carotid artery (ECA) were measured at predefined plaque-free segments. The carotid bulb diameter was defined as the maximum luminal diameter at the most proximal segment of the ICA. All diameters were measured perpendicular to the vessel axis.

All measurements were performed bilaterally according to a predefined acquisition protocol. Optimal visualization of the CB and ICA was achieved when the vessel could be demonstrated as a continuous structure extending from the CCA, as illustrated in Figures 1A,C,D. Vessel diameters were measured at end-diastole from media to media in plaque-free segments with parallel vessel walls (Figure 1B), using a single standardized measurement per arterial segment. The CCA diameter was measured in the distal segment proximal to the bifurcation (Figure 1E). ICA diameters were measured in the proximal segment immediately distal to the CB, i.e., at the beginning of the uniform diameter of the ICA, ECA diameters were obtained in the proximal ECA segment distal to the carotid bifurcation. The CB diameter was defined as the largest diameter at the most proximal segment of the ICA, as schematically illustrated in Figure 2.

Vessels with local luminal narrowing exceeding 20% were excluded. In addition, vessels were excluded if CB could not be clearly delineated. Also, subjects exhibiting a CB starting at the distal CCA or predominantly in the ECA were not considered.

To ensure consistency and measurement accuracy, vessel diameters were reassessed offline in a subset of examinations using stored DICOM images. Offline measurements followed the identical predefined protocol applied during real-time acquisition and were performed prior to the statistical analysis. Offline re-measurement served solely for quality control and was not triggered by study outcomes.

### Measures to reduce inter-center variability

To minimize potential center and device-related variability, all examinations were performed according to a standardized acquisition protocol using predefined anatomical landmarks. Ratio-based geometric parameters were selected *a priori* as primary outcome measures to reduce the influence of absolute diameter differences between ultrasound systems. Measurement consistency was further supported by intra-rater reliability assessment based on repeat offline measurements in a randomly predefined subset of examinations; detailed results are provided in the Supplementary material.

### Statistical analysis

Baseline characteristics of the study population were summarized descriptively and stratified by Ethnicity (White vs. Asian). Continuous variables are presented as mean ± standard deviation, and categorical variables as counts and percentages. In accordance with current recommendations for observational studies, no formal statistical hypothesis testing was performed for baseline comparisons, as group membership was defined as *a priori* by ethnicity rather than by randomization.

Carotid bifurcation geometry was quantified using predefined diameter ratios, including CB to ICA (CB/ICA), CB to CCA (CB/CCA), ICA to CCA (ICA/CCA), ECA to CCA (ECA/CCA), ECA to ICA (ECA/ICA), and the outflow/inflow ratio. Linear mixed-effects models were applied to account for within-subject correlation due to bilateral measurements. This approach preserves statistical efficiency and avoids information loss compared with averaging measurements.

In the primary analysis, ethnicity was included as a fixed effect, while bilateral measurements (left and right side) were modeled as repeated observations within individuals, with subject identifier specified as a random intercept. Estimated marginal means (EMMs) with 95% confidence intervals (CIs) were derived for each geometry ratio by ethnicity. Models were fitted using restricted maximum likelihood (REML) estimation, with degrees of freedom calculated using the Satterthwaite approximation.

For the fully adjusted analysis, models were additionally adjusted for age, sex, body mass index (BMI), diabetes mellitus, hypertension, dyslipidemia, smoking status, and atrial fibrillation. An unstructured covariance matrix was specified for repeated measurements within individuals. Adjusted mean differences between ethnic groups (Asian minus White) with corresponding 95% CIs and *p*-values were reported. A two-sided p-value < 0.05 was considered statistically significant.

Model assumptions for the primary outcome (CB/ICA) were assessed by inspection of standardized residuals, Q–Q plots, and residuals versus fitted values. No relevant deviations from normality or homoscedasticity were observed, and no transformation of outcome variables was required (Supplementary Figures S1–S3).

Additional sensitivity analyses using alternative covariance structures, intra-rater reliability assessed by intraclass correlation coefficients (ICC), and model diagnostic assessments are reported in the Supplementary material.

Inferential statistical analyses were performed using IBM SPSS Statistics, version 26 (IBM Corp., Armonk, NY, United States). Descriptive analyses and graphical visualizations were generated using R (R Foundation for Statistical Computing, Vienna, Austria) and RStudio (Posit, PBC, Boston, MA, United States).

## Results

A total of 200 participants were included, comprising 100 White participants (50 women and 50 men) and 100 Asian participants (50 women and 50 men). Baseline demographic characteristics and cardiovascular risk factor profiles are presented in Table 1. White participants were older on average (56.09 ± 18.98 vs. 43.41 ± 12.88 years) and exhibited a higher prevalence of cardiovascular risk factors, including diabetes mellitus (16% vs. 5%), hypertension (50% vs. 20%), dyslipidemia (45% vs. 28%), smoking (31% vs. 18%), and atrial fibrillation (8% vs. 0%). These variables were considered potential confounders and were therefore included as covariates in the adjusted analyses.

**Table 1:** Baseline characteristics of the study population are stratified by ethnicity.

Variable	Total (*n* = 200)	White (*n* = 100)	Asian (*n* = 100)
Demographics			
Age, years	49.75 ± 17.38	56.09 ± 18.98	43.41 ± 12.88
Female sex, *n* (%)	100 (50.0)	50 (50.0)	50 (50.0)
Body mass index, kg/m^2^	26.27 ± 4.62	27.33 ± 4.64	25.20 ± 4.37
Cardiovascular risk factors			
Diabetes mellitus, *n* (%)	21 (10.5)	16 (16.0)	5 (5.0)
Hypertension, *n* (%)	70 (35.0)	50 (50.0)	20 (20.0)
Dyslipidemia, *n* (%)	73 (36.5)	45 (45.0)	28 (28.0)
Smoking, *n* (%)	49 (24.5)	31 (31.0)	18 (18.0)
Atrial fibrillation, *n* (%)	8 (4.0)	8 (8.0)	0 (0.0)

Values are presented as mean ± standard deviation or number (percentage). No statistical testing was performed for baseline comparisons.

Prior to the main statistical analyses, intraobserver reproducibility was assessed using intraclass correlation coefficients (ICC) based on a two-way mixed-effects model with absolute agreement. Repeated measurements were performed in a subset of participants (*n* = 30), and reliability was evaluated separately for left and right sides. Formal interobserver reproducibility analyses were not performed in the present study.

Intraobserver reliability was excellent across all measured vessel segments. For CB diameter measurements, single-measure ICCs were 0.97 (95% CI, 0.95–0.99) on the left side and 0.99 (95% CI, 0.98–0.99) on the right side. For CCA diameter, corresponding ICCs were 0.98 (95% CI, 0.95–0.98) on the left and 0.98 (95% CI, 0.96–0.99) on the right. Internal carotid artery diameter measurements likewise demonstrated high reliability, with ICCs of 0.97 (95% CI, 0.94–0.98) on the left and 0.96 (95% CI, 0.91–0.98) on the right. Interobserver reliability was not formally assessed, as all measurements were performed according to a standardized protocol and supervised by an experienced investigator; however, this should be addressed in future studies.

Estimated marginal means derived from linear mixed-effects models before adjustment for potential confounders demonstrated significant ethnicity differences across all primary carotid geometry ratios (Table 2). The CB/ICA ratio, defined *a priori* as the primary outcome, was significantly lower in Asian participants compared with White participants (EMM 1.57 vs. 1.68; *p* < 0.001). In contrast, Asian participants exhibited higher CB/CCA (EMM 1.27 vs. 1.20; *p* < 0.001) and ICA/CCA ratios (EMM 0.82 vs. 0.72; p < 0.001), indicating relatively larger distal vessel calibers in relation to the CCA. The outflow/inflow ratio was also higher in Asian participants (EMM 1.16 vs. 0.96; *p* < 0.001), whereas the ECA/ICA ratio was lower (EMM 0.84 vs. 0.92; *p* < 0.001), reflecting systematic differences in carotid bifurcation geometry between both groups.

**Table 2:** Model-based estimated marginal means of carotid bifurcation geometry ratios by ethnicity (ethnicity-only linear mixed models).

Geometry ratio	White (EMM, 95% CI)	Asian (EMM, 95% CI)	*p*-value
CB/ICA	1.68 (1.65–1.71)	1.57 (1.54–1.60)	<0.001
CB/CCA	1.20 (1.18–1.23)	1.28 (1.25–1.30)	<0.001
ICA/CCA	0.72 (0.70–0.74)	0.82 (0.81–0.84)	<0.001
ECA/ICA	0.92 (0.90–0.4)	0.84 (0.82–0.86)	<0.001
ECA/CCA	0.66 (0.64–0.67)	0.68 (0.67–0.70)	0.030
Outflow/Inflow	0.97 (0.93–1.01)	1.17 (1.13–1.21)	<0.001

Values are estimated marginal means (EMM) with 95% confidence intervals (CI) derived from linear mixed models accounting for repeated left–right measurements within individuals. CB, carotid bulb; ICA, internal carotid artery; CCA, common carotid artery; ECA, external carotid artery; inflow/outflow ratio is calculated as (ICA^2^ + ECA^2^)/CCA^2^.

**Figure 3:**
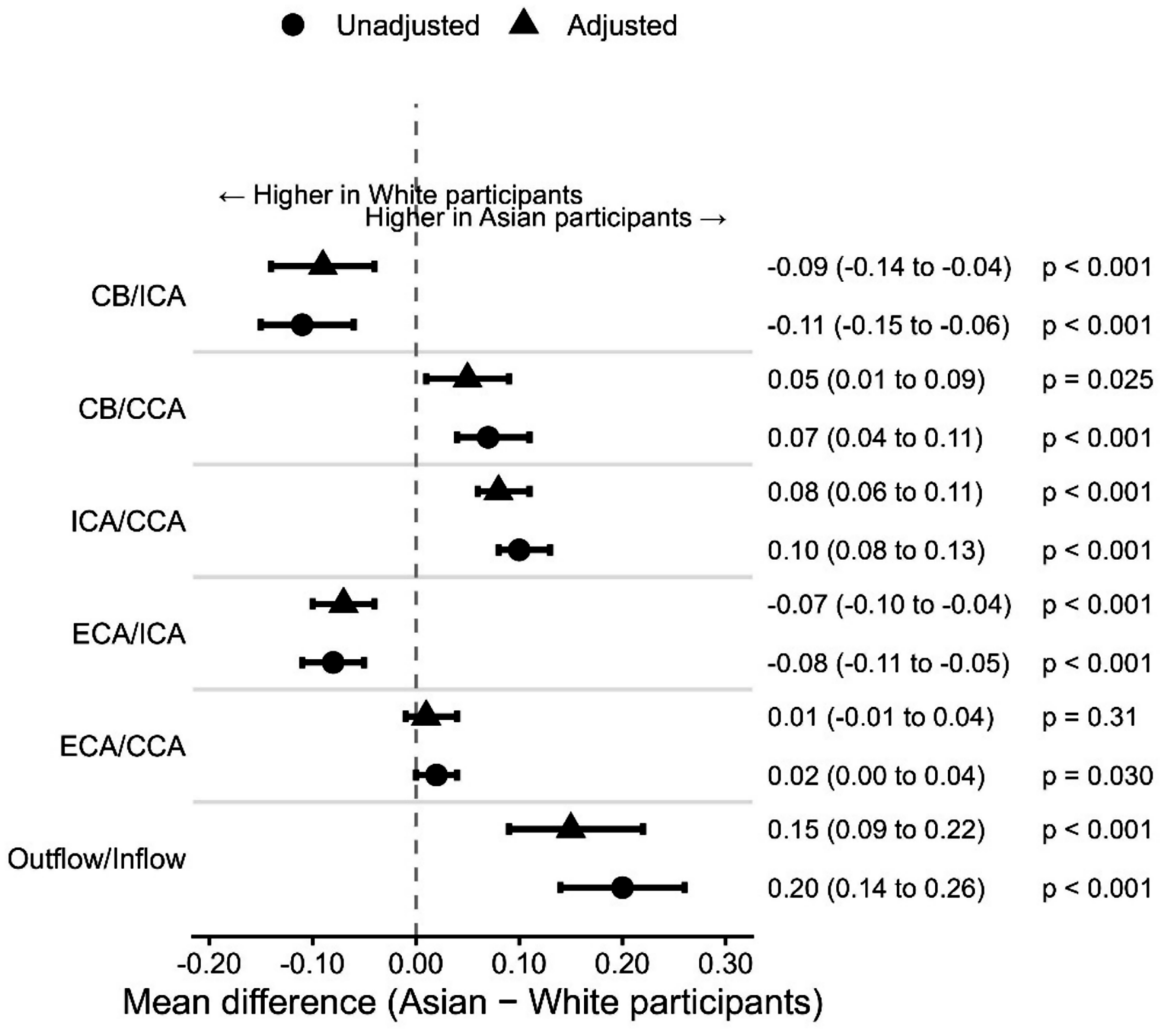
Summary of the magnitude and direction of ethnicity-associated differences in carotid bifurcation geometry ratios before and after multivariable adjustment. Forest plot showing mean differences (Asian – White) with 95% confidence intervals for carotid bifurcation geometry ratios. Circles represent estimates from ethnicity-only linear mixed-effects models (unadjusted), and triangles represent estimates from fully adjusted models. Fully adjusted models included age, sex, body mass index, diabetes mellitus, hypertension, dyslipidemia, smoking status, and atrial fibrillation as fixed effects. Repeated left–right measurements within individuals were accounted for using a linear mixed-effects modeling framework. Positive values indicate higher ratios in Asian participants, whereas negative values indicate higher ratios in White participants. CB, carotid bulb; ICA, internal carotid artery; CCA, common carotid artery; ECA, external carotid artery. The outflow/inflow ratio was calculated as (ICA^2^ + ECA^2^)/CCA^2^.

After multivariable adjustment, the observed ethnic differences in carotid bifurcation geometry remained largely unchanged (Table 3, Figure 3). The adjusted mean difference in CB/ICA (Asian minus White participants) was −0.09 (95% CI −0.14 to −0.04; *p* < 0.001). The CB/CCA and ICA/CCA ratios remained significantly higher in Asian participants, whereas the ECA/ICA ratio remained significantly lower. The outflow/inflow area ratio demonstrated a robust positive adjusted difference (0.15; 95% CI 0.09 to 0.22; *p* < 0.001). In contrast, no statistically significant racial difference was observed for the ECA/CCA ratio after multivariable adjustment.

**Table 3:** Association between ethnicity and carotid bifurcation geometry ratios assessed by linear mixed-effects models.

Geometry ratio	Adjusted mean difference (Asian-White group)	95% CI	*p*-value
CB/ICA	−0.09	−0.14 to −0.04	<0.001
CB/CCA	0.05	0.01 to 0.09	0.025
ICA/CCA	0.08	0.06 to 0.11	<0.001
ECA/ICA	−0.07	−0.10 to −0.04	<0.001
ECA/CCA	0.01	−0.01 to 0.07	0.31
Outflow/Inflow	0.15	0.09 to 0.22	<0.001

Adjusted mean differences (*β*) of Linear mixed-effects models with ethnicity as fixed effect; adjusted for age, sex, body mass index, diabetes mellitus, hypertension, dyslipidemia, smoking, and atrial fibrillation. Repeated measures by side within subject using a compound symmetry covariance matrix. REML estimation; Satterthwaite degrees of freedom; 95% confidence intervals. CB, carotid bulb; ICA, internal carotid artery; CCA, common carotid artery; ECA, external carotid artery; outflow/inflow area ratio is calculated as (ICA^2^ + ECA^2^)/CCA^2^.

Full covariate-specific estimates from the fully adjusted linear mixed-effects models for each carotid geometry ratio are provided in Supplementary Tables S1–S6.

Sensitivity analyses confirmed the robustness of the primary findings across alternative model specifications. To explore which vascular segment primarily contributed to the observed CB/ICA ratio differences, absolute vessel diameters were analyzed as separate outcomes using otherwise identical mixed-effects models. These analyses demonstrated no significant ethnic difference in absolute ICA diameter, whereas CB diameter was significantly smaller in Asian participants. This pattern indicates that the lower CB/ICA ratio observed in Asian participants was primarily driven by differences in CB geometry rather than by variation in ICA caliber. Corresponding results are summarized in Supplementary Table S7.

For descriptive and exploratory purposes, unadjusted absolute vessel diameters of the CCA, CB, ICA, and ECA were summarized by ethnicity and are provided in the Supplementary material, both as side-averaged values per participant (Supplementary Table S8) and as side-specific values (Supplementary Table S9) and are demonstrated graphically in Supplementary Figures S4, S5.

## Discussion

In this cross-sectional ultrasound study, we identified consistent ethnic differences in carotid bifurcation geometry between Asian and White adults. Asian participants exhibited a lower CB/ICA ratio, along with higher ICA/CCA ratio and outflow/inflow area ratios, indicating a proportionally smaller CB relative to downstream vessels. These differences were observed in both unadjusted and adjusted analyses, suggesting they are not explained by conventional vascular risk factors.

These findings suggest that anatomical variation in carotid bifurcation geometry may contribute to population-specific hemodynamic environments and could influence the localization and development of atherosclerotic disease. This may have implications for individualized cerebrovascular risk assessment and for understanding population differences in stroke patterns.

Prior studies have shown that proportional relationships between the common, internal, and external carotid arteries influence flow separation and WSS at the carotid bifurcation (11, 12). Variability in carotid bifurcation geometry has been documented using ultrasound and angiographic techniques (7, 13, 14). Interethnic differences in carotid bifurcation ratios have also been reported in angiographic studies (15), supporting the concept that proportional carotid anatomy varies across populations. However, these investigations were largely conducted in stroke populations and did not specifically focus on CB morphology, and disease-related vascular remodeling may have influenced the findings. In contrast, the present study assessed proportional carotid geometry in individuals without evidence of carotid stenosis, providing a largely disease-free anatomical context. Therefore, whether population-level differences in carotid disease distribution relate to underlying differences in CB geometry remains uncertain.

By applying a ratio-based approach to two ethnically distinct populations examined using a standardized ultrasound protocol, the present study extends existing literature by demonstrating that ethnic differences in carotid bifurcation anatomy are predominantly proportional and primarily driven by variation in CB morphology rather than by absolute ICA diameter. This pattern is particularly relevant given prior evidence linking cross-sectional area ratios at the carotid bifurcation to atherosclerotic plaque development (16, 17).

From a pathophysiological perspective, the observed differences in proportional carotid geometry may provide a structural context for previously reported ethnic variation in the distribution of carotid and cerebrovascular atherosclerosis. A comparatively small CB relative to the downstream vessels may change blood flow patterns at the bifurcation. This could influence areas with low or fluctuating WSS, which are known to be more prone to atherosclerotic changes. However, these results should be considered exploratory, since we did not directly examine plaque burden or clinical outcomes.

Several limitations should be acknowledged. First, the cross-sectional design precludes causal inference, and causal relationships between carotid geometry and vascular disease cannot be established; therefore, the observed associations should be interpreted as hypothesis-generating. The two study populations differed in baseline characteristics, including age and vascular risk factors. Although statistical adjustment was performed, residual confounding cannot be excluded. Second, the analysis focused on geometric parameters and did not include direct assessment of carotid plaque burden, intima-media thickness, stenosis severity, or cerebrovascular outcomes. Third, ethnicity was defined by self-report and may not fully capture genetic, environmental, or lifestyle heterogeneity within groups that could contribute to vascular anatomical variation. Fourth, participants were recruited from one center per ethnicity group, and although identical examination protocols were applied, center-specific factors related to recruitment or clinical setting cannot be fully excluded, which may limit generalizability to similar populations studied in other geographic or clinical settings. In addition, the proportions of participants recruited as inpatients, outpatients, and healthy volunteers were not systematically recorded and could therefore not be analyzed.

Fifth, interobserver reliability was not formally assessed, as measurements were performed according to a standardized protocol and were subject to centralized quality control by an experienced investigator; however, this remains a limitation.

Finally, although the observed proportional differences were statistically robust, their magnitude was modest, and their clinical relevance remains to be established; in addition, cardiovascular risk factors such as physical activity, an important modifiable cardiovascular risk factor (18, 19), were not assessed and could therefore not be included as covariates; however, the direct influence of physical activity on carotid bifurcation geometry remains uncertain. In addition, heart rate at the time of examination was not recorded and could therefore not be considered as a potential covariate.

In conclusion, this cross-sectional ultrasound study demonstrates systematic ethnicity differences in carotid bifurcation geometry between Asian and White adults. These differences are predominantly proportional and appear to be driven by variation in CB morphology rather than by absolute ICA diameter. Although the clinical implications remain to be established, the findings highlight CB geometry as a population-specific anatomical feature that may be relevant for the formation of carotid plaques across populations. A large CB could therefore be considered a risk factor for the development of plaques. Future studies integrating detailed hemodynamic assessment, including computational fluid dynamics, vascular wall pathology, and longitudinal clinical outcomes are warranted to clarify the significance of these geometric differences.

## Data Availability

The raw data supporting the conclusions of this article will be made available by the authors, without undue reservation.
