# Supplementary material for: Ethnic differences in carotid bulb geometry between Asian and White populations
Source: Front Neurol. 2026 Apr 21;17:1805941. doi: 10.3389/fneur.2026.1805941 (PMC13141853; doi:10.3389/fneur.2026.1805941)
Supplement: Supplementary file 1 [file Data_Sheet_1.pdf]

Supplementary Introduction

Rationale for Selection of the Carotid Bulb-to-Internal Carotid Artery (CB/ICA) Ratio as the Primary Outcome

The present study was designed to investigate interethnic differences in carotid artery geometry with a particular focus on CB. Because no single geometric parameter has been universally accepted to characterize CB morphology, careful a priori selection of the primary outcome measure was essential to ensure anatomical relevance, physiological interpretability, and methodological robustness.

Anatomical and Embryological Considerations

The CB represents a physiological dilation located at the proximal segment of the ICA and constitutes a transitional zone between the extracranial inflow and the cerebral circulation. Although the common carotid artery (CCA) and ICA form a continuous structure in adult anatomy, they differ in their embryological contributions. The ICA develops predominantly from the third aortic arch and the cranial portion of the dorsal aorta, whereas the CCA is derived mainly from the ventral aortic sac and proximal segments of the third aortic arch[1]. These differences in embryological contribution are associated with regional heterogeneity in arterial wall composition and adaptive behavior, which persists in adult life and may influence vascular remodeling responses [2,3].

Hemodynamic and Systemic Influences on CCA Versus ICA Diameter

From a functional perspective, the CCA primarily serves as an elastic conduit vessel transmitting cardiac output toward the carotid bifurcation. Its diameter has been shown to scale with systemic hemodynamic load and anthropometric factors, including age, blood pressure, body size, and arterial stiffness, even in healthy populations [4–6]. Therefore, CCA diameter exhibits substantial interindividual and interpopulation variability.

In contrast, the ICA represents the principal inflow vessel to the anterior cerebral circulation, and its caliber has been shown to relate to cerebral perfusion. Although ICA diameter is not invariant and may also be influenced by systemic factors, it constitutes the

immediate downstream vessel of the CB and therefore provides a physiologically relevant local reference when characterizing bulb-related geometry.

Relationship Between the ICA and Intracranial Circulation

The ICA is functionally coupled with the intracranial arterial tree and supplies most cerebral blood flow to the anterior circulation. Prior imaging and flow studies have demonstrated that the internal carotid arteries constitute the principal extracranial inflow pathways supplying total cerebral blood flow[7]. In this context, the CB may be regarded as a flow-conditioning structure whose geometric relationship to the ICA is directly relevant to downstream cerebral hemodynamics.

Justification for CB/ICA Over CB/CCA

Although the CB/CCA ratio captures bulb size relative to the inflow vessel, it may obscure local bulb morphology by incorporating upstream arterial scaling driven by systemic and anthropometric influences. Given known interethnic differences in body habitus and cardiovascular risk profiles. In contrast, the CB/ICA ratio should isolate the relationship between the bulb and its immediate outflow tract and is therefore less susceptible to confounding by generalized vessel size.

Rationale for Ratio-Based Metrics and Analytical Hierarchy

Absolute vessel diameters are inherently scale-dependent and strongly correlated with anthropometric variables. Ratio-based metrics normalize for interindividual variability in overall vessel size and facilitate more meaningful comparisons of relative vascular geometry between populations. Accordingly, the CB/ICA ratio was defined a priori as the primary anatomical outcome of interest. Other carotid geometry ratios, including CB/CCA and ICA/CCA, were prespecified as secondary outcomes intended to provide complementary anatomical context. Sensitivity analyses using absolute diameter models were performed to assess the robustness of the primary findings but were not intended to redefine the primary hypothesis.

This Supplementary Introduction provides additional methodological context for the priori selection of the primary outcome

Supplementary Methods

Descriptive Analysis of Absolute Diameters

Absolute vessel diameters were analyzed descriptively only. Descriptive summaries were generated using two approaches: (1) subject-level averages derived from bilateral measurements (Figure S1) and (2) side-specific summaries comparing left and right measurements (Figure S2). Absolute diameters were not entered into inferential mixed-effects models.

Assessment of Model Assumptions and Diagnostics

Model assumptions for the primary linear mixed-effects model with the CB/ICA ratio as primary outcome were evaluated using standard residual-based diagnostic procedures.

Residual normality was assessed by visual inspection of the distribution of standardized residuals, including histograms and normal quantile–quantile (Q–Q) plots. Standardized residuals were approximately normally distributed, with a mean close to zero and a standard deviation close to one. The histogram was symmetric, and the Q–Q plot demonstrated close adherence of the residuals to the theoretical normal distribution, indicating acceptable residual normality (Figures S3-S4)

Homoscedasticity was evaluated by plotting standardized residuals against fitted values. Residuals were randomly dispersed around zero without evidence of funneling, curvature, or other systematic patterns, supporting the assumption of constant variance across the range of fitted values (Figure S5)

Potential influential observations were examined using standardized residual range checks. Standardized residuals ranged from approximately -2.36 to +2.92, with no observations exceeding ±3. This distribution did not suggest the presence of extreme outliers or influential data points dominating model estimates.

The need for outcome transformation was considered as part of the diagnostic evaluation. Given the acceptable residual normality, homoscedasticity, and absence of influential outliers, transformation of the outcome variable was deemed unnecessary and was not performed.

Overall, diagnostic assessments indicated that the assumptions of the linear mixed-effects model for the primary CB/ICA outcome were adequately met.

Supplementary Tables

Table S1. Fixed-effect estimates from linear mixed-effects models for the carotid bulb to internal carotid artery ratio (CB/ICA)

			95% Confidence Interval	
**Parameter**	**β estimates**	**p-value**	**Lower Bound**	**Upper Bound**
Asian-White	-0.09	<0.001	-0.14	-0.04
Female vs male	0.03	0.234	-0.02	0.07
Diabetes mellitus	0.11	0.005	0.03	0.18
[Hypertension	-0.07	0.017	-0.12	-0.01
Dyslipidemia	-0.01	0.727	-0.06	0.04
Smoking	0.05	0.063	0.00	0.08
Atrial fibrillation	0.03	0.637	-0.08	0.14
Age	0.00	0.070	0.00	0.00
BMI	0.00	0.861	-0.01	0.01

β estimates represent fixed effects derived from fully adjusted linear mixed-effects models. Models included ethnicity, age, sex, body mass index, diabetes mellitus, hypertension, dyslipidemia, smoking status, atrial fibrillation as fixed effects. Bilateral measurements were modelled as repeated observations within subjects. Restricted maximum likelihood estimation with Satterthwaite approximation for degrees of freedom was used.

For binary covariates, β estimates represent the comparison category relative to the reference category (e.g., female vs male; present vs absent). For continuous covariates, β estimates represent the expected change in the outcome per one-unit increase.

β estimates and Confidence intervals are rounded to two decimal places. P-values are reported to three decimals

Table S2. Fixed-effect estimates from linear mixed-effects models for the carotid bulb to common carotid artery ratio (CB/CCA)

			95% Confidence Interval	
**Parameter**	**β estimates**	**p-value**	**95% CI (Lower)**	**95% CI (Upper)**
Asian-Whie	0.05	0.025	0.01	0.08
Female vs male	0.00	0.916	-0.04	0.04
Diabetes mellitus	0.01	0.632	-0.05	0.08
Hypertension	-0.08	0.001	-0.13	-0.04
Dyslipidemia	0.01	0.617	-0.03	0.05
Smoking	0.02	0.296	-0.02	0.06
Atrial fibrillation	-0.01	0.821	-0.10	0.08
Age	0.00	0.395	0.00	0.00
BMI	0.00	0.798	0.00	0.00

β estimates represent fixed effects derived from fully adjusted linear mixed-effects models. Models included ethnicity, age, sex, body mass index, diabetes mellitus, hypertension, dyslipidemia, smoking status, atrial fibrillation as fixed effects. Bilateral measurements were modelled as repeated observations within subjects. Restricted maximum likelihood estimation with Satterthwaite approximation for degrees of freedom was used. For binary covariates, β estimates represent the comparison category relative to the reference category (e.g., female vs male; present vs absent). For continuous covariates, β estimates represent the expected change in the outcome per one-unit increase. β estimates and confidence intervals are rounded to two decimal places. P-values are reported to three decimals. Values with absolute magnitude <0.005 are shown as 0.00.

Table S3. Fixed-effect estimates from linear mixed-effects models for the internal carotid artery to common carotid artery ratio (ICA/CCA)

			95% Confidence Interval	
**Parameter**	**β estimates**	**p-value**	**95% CI (Lower)**	**95% CI (Upper)**
Asian-White	0.08	<0.001	0.05	0.11
Female vs male	-0.01	0.349	-0.04	0.01
Diabetes mellitus	-0.04	0.063	-0.08	0.00
Hypertension	-0.02	0.285	-0.05	0.01
Dyslipidemia	0.00	0.756	-0.02	0.03
Smoking	-0.01	0.698	-0.03	0.02
Atrial fibrillation	-0.02	0.631	-0.08	0.05
Age	0.00	0.084	0.00	0.00
BMI	0.00	0.805	0.00	0.00

β estimates represent fixed effects derived from fully adjusted linear mixed-effects models. Models included ethnicity, age, sex, body mass index, diabetes mellitus, hypertension, dyslipidemia, smoking status, atrial fibrillation as fixed effects. Bilateral measurements were modelled as repeated observations within subjects. Restricted maximum likelihood estimation with Satterthwaite approximation for degrees of freedom was used.

For binary covariates, β estimates represent the comparison category relative to the reference category (e.g., female vs male; present vs absent). For continuous covariates, β estimates represent the expected change in the outcome per one-unit increase.

β estimates and confidence intervals are rounded to two decimal places. P-values are reported to three decimals. Values with absolute magnitude <0.005 are shown as 0.00.

Table S4. Fixed-effect estimates from linear mixed-effects models for the outflow-to-inflow ratio at the carotid bifurcation

			95% Confidence Interval	
**Parameter**	**β estimates**	**p-value**	**95% CI (Lower)**	**95% CI (Upper)**
Asian-White	0.15	<0.001	0.09	0.22
Female vs male	-0.05	0.090	-0.12	0.01
Diabetes mellitus	-0.09	0.082	-0.19	0.01
Hypertension	-0.05	0.210	-0.13	0.03
Dyslipidemia	0.01	0.688	-0.05	0.08
Smoking	-0.02	0.584	-0.09	0.05
Atrial fibrillation	0.01	0.878	-0.14	0.16
Age	0.00	0.064	-0.00	0.00
BMI	0.00	0.697	-0.01	0.01

β estimates represent fixed effects derived from fully adjusted linear mixed-effects models. Models included ethnicity, age, sex, body mass index, diabetes mellitus, hypertension, dyslipidemia, smoking status, atrial fibrillation as fixed effects. Bilateral measurements were modelled as repeated observations within subjects. Restricted maximum likelihood estimation with Satterthwaite approximation for degrees of freedom was used. For binary covariates, β estimates represent the comparison category relative to the reference category (e.g., female vs male; present vs absent). For continuous covariates, β estimates represent the expected change in the outcome per one-unit increase.

β estimates and confidence intervals are rounded to two decimal places. P-values are reported to three decimals. Values with absolute magnitude <0.005 are shown as 0.00.

Table S5. Fixed-effect estimates from linear mixed-effects models for the external carotid artery to common carotid artery ratio (ECA/CCA)

			95% Confidence Interval	
**Parameter**	**β estimates**	**p-value**	**95% CI (Lower)**	**95% CI (Upper)**
Asian-White	0.01	0.310	-0.01	0.04
Female vs male	-0.02	0.050	-0.05	0.00
Diabetes mellitus	-0.02	0.354	-0.05	0.02
Hypertension	-0.02	0.221	-0.05	0.01
Dyslipidemia	0.01	0.551	-0.02	0.03
Smoking	-0.01	0.434	-0.04	0.02
Atrial fibrillation	0.02	0.397	-0.03	0.08
Age	0.00	0.055	0.00	0.00
BMI	0.00	0.244	0.00	0.00

β estimates represent fixed effects derived from fully adjusted linear mixed-effects models. Models included ethnicity, age, sex, body mass index, diabetes mellitus, hypertension, dyslipidemia, smoking status, atrial fibrillation as fixed effects. Bilateral measurements were modelled as repeated observations within subjects. Restricted maximum likelihood estimation with Satterthwaite approximation for degrees of freedom was used.

For binary covariates, β estimates represent the comparison category relative to the reference category (e.g., female vs male; present vs absent). For continuous covariates, β estimates represent the expected change in the outcome per one-unit increase.

β estimates and confidence intervals are rounded to two decimal places. P-values are reported to three decimals. Values with absolute magnitude <0.005 are shown as 0.00.

Table S6. Fixed-effect estimates from linear mixed-effects models for the external carotid artery to internal carotid artery ratio (ECA/ICA)

			95% Confidence Interval	
**Parameter**	**β Estimate**	**p-value**	**95% CI (Lower)**	**95% CI (Upper)**
Asian-White	-0.07	0.000	-0.10	-0.04
Female vs male	-0.01	0.392	-0.05	0.02
Diabetes mellitus	0.03	0.320	-0.03	0.08
Hypertension	0.00	0.917	-0.04	0.04
Dyslipidemia	0.00	0.901	-0.03	0.04
Smoking	-0.01	0.743	-0.04	0.03
Atrial fibrillation	0.06	0.153	-0.02	0.13
Age	0.00	0.878	0.00	0.00
BMI	0.00	0.218	0.00	0.01

β estimates represent fixed effects derived from fully adjusted linear mixed-effects models. Models included ethnicity, age, sex, body mass index, diabetes mellitus, hypertension, dyslipidemia, smoking status, atrial fibrillation as fixed effects. Bilateral measurements were modelled as repeated observations within subjects. Restricted maximum likelihood estimation with Satterthwaite approximation for degrees of freedom was used.

For binary covariates, β estimates represent the comparison category relative to the reference category (e.g., female vs male; present vs absent). For continuous covariates, β estimates represent the expected change in the outcome per one-unit increase.

β estimates and confidence intervals are rounded to two decimal places. P-values are reported to three decimals. Values with absolute magnitude <0.005 are shown as 0.00.

Table S7. Sensitivity analysis of the association between ethnicity and carotid bulb–to–internal carotid artery (CB/ICA) ratio using denominator-adjusted and absolute diameter mixed-effects models.

Outcome	Model	Ethnicity β (Asian–White Participant)	95% CI	p-value
CB/ICA	Primary LMM	-0.09	-0.14 to -0.04	< 0.001
ICA	Absolute	0.08	-0.09 to 0.24	0.354
CB	Absolute	-0.43	-0.66 to -0.21	<0.001

Values represent adjusted β coefficients (Asian – White) derived from linear mixed-effects models (LMM) with subject-specific random intercepts and repeated left-right measurements. Absolute diameter models used internal carotid artery or carotid bulb diameter as the dependent variable. All models were adjusted for age, sex, body mass index, diabetes mellitus, hypertension, dyslipidemia, smoking status, atrial fibrillation.

CB = carotid bulb; ICA = internal carotid artery; CCA = common carotid artery.

Table S8. Carotid arteries Raw diameters by ethnicity

*Values are presented as mean ± standard deviation (mm) and are provided for descriptive purposes only***.**

**Measurement**	**Asian (Mean ± SD, mm)**	**White (Mean ± SD, mm)**
Common carotid artery (CCA)	6.79 ± 0.68	7.90 ± 1.06
Carotid bulb	8.65 ± 1.03	9.41 ± 1.24
Internal carotid artery (ICA)	5.58 ± 0.86	5.65 ± 0.72
External carotid artery (ECA)	4.61 ± 0.66	5.15 ± 0.81

Values are presented as mean ± standard deviation (mm) and are provided for descriptive purposes only. No formal hypothesis tests were performed.

Table S9. Carotid arteries Raw diameters by ethnicity and side

Values are presented as mean ± standard deviation (mm) and are provided for descriptive purposes only.

Measurement	Side	Asian (Mean ± SD, mm)	White (Mean ± SD, mm)
Common carotid artery (CCA)	Right	6.78 ± 0.69	7.88 ± 1.07
Common carotid artery (CCA)	Left	6.80 ± 0.68	7.92 ± 1.05
Carotid bulb	Right	8.64 ± 1.04	9.39 ± 1.25
Carotid bulb	Left	8.66 ± 1.02	9.43 ± 1.23
Internal carotid artery (ICA)	Right	5.56 ± 0.86	5.63 ± 0.73
Internal carotid artery (ICA)	Left	5.60 ± 0.87	5.67 ± 0.71
External carotid artery (ECA)	Right	4.60 ± 0.66	5.14 ± 0.82
External carotid artery (ECA)	Left	4.62 ± 0.65	5.16 ± 0.80

Values are presented as mean ± standard deviation (mm) and are provided for descriptive purposes only. No formal hypothesis tests were performed.

Supplementary Figures

Figure S1

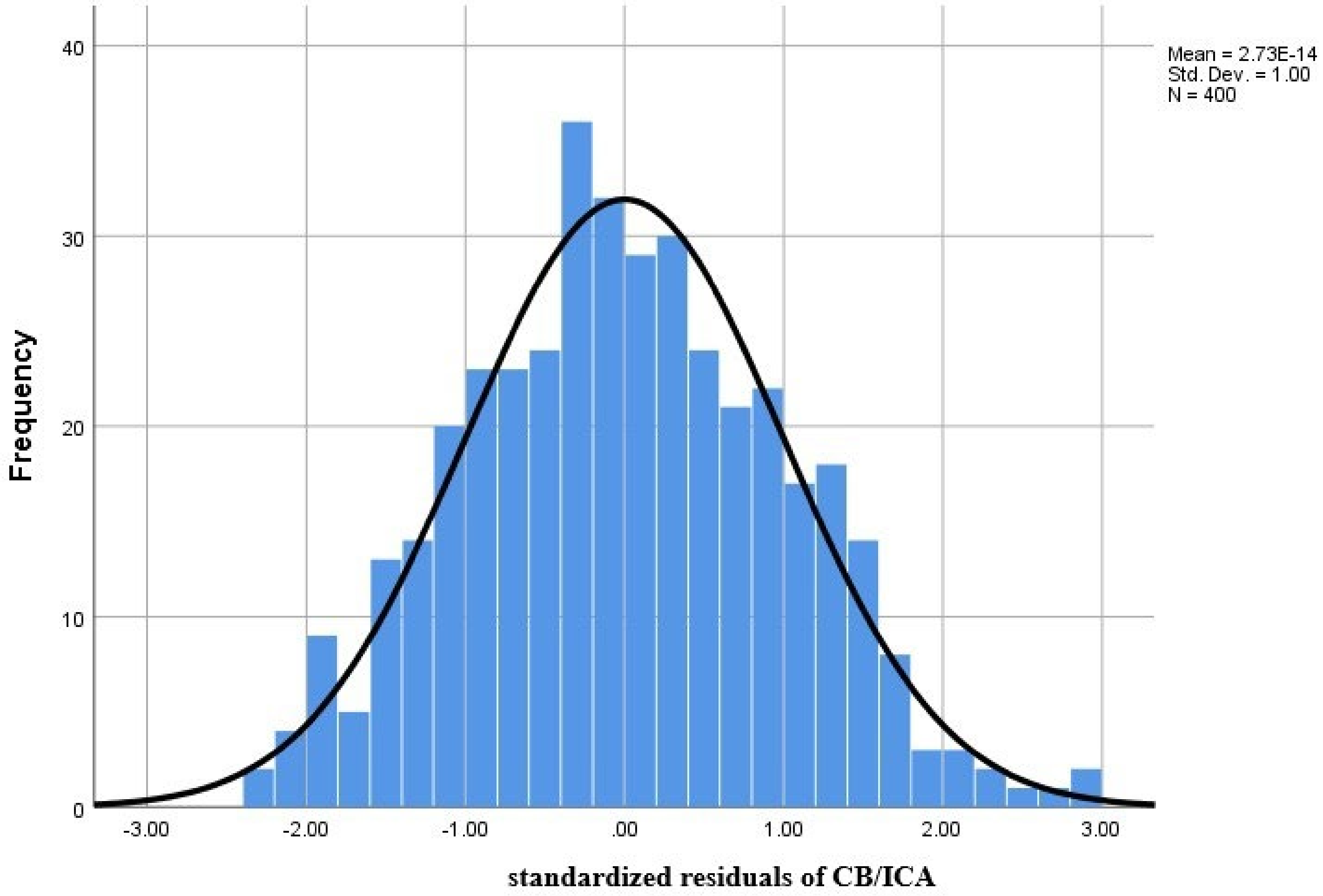

Figure S2

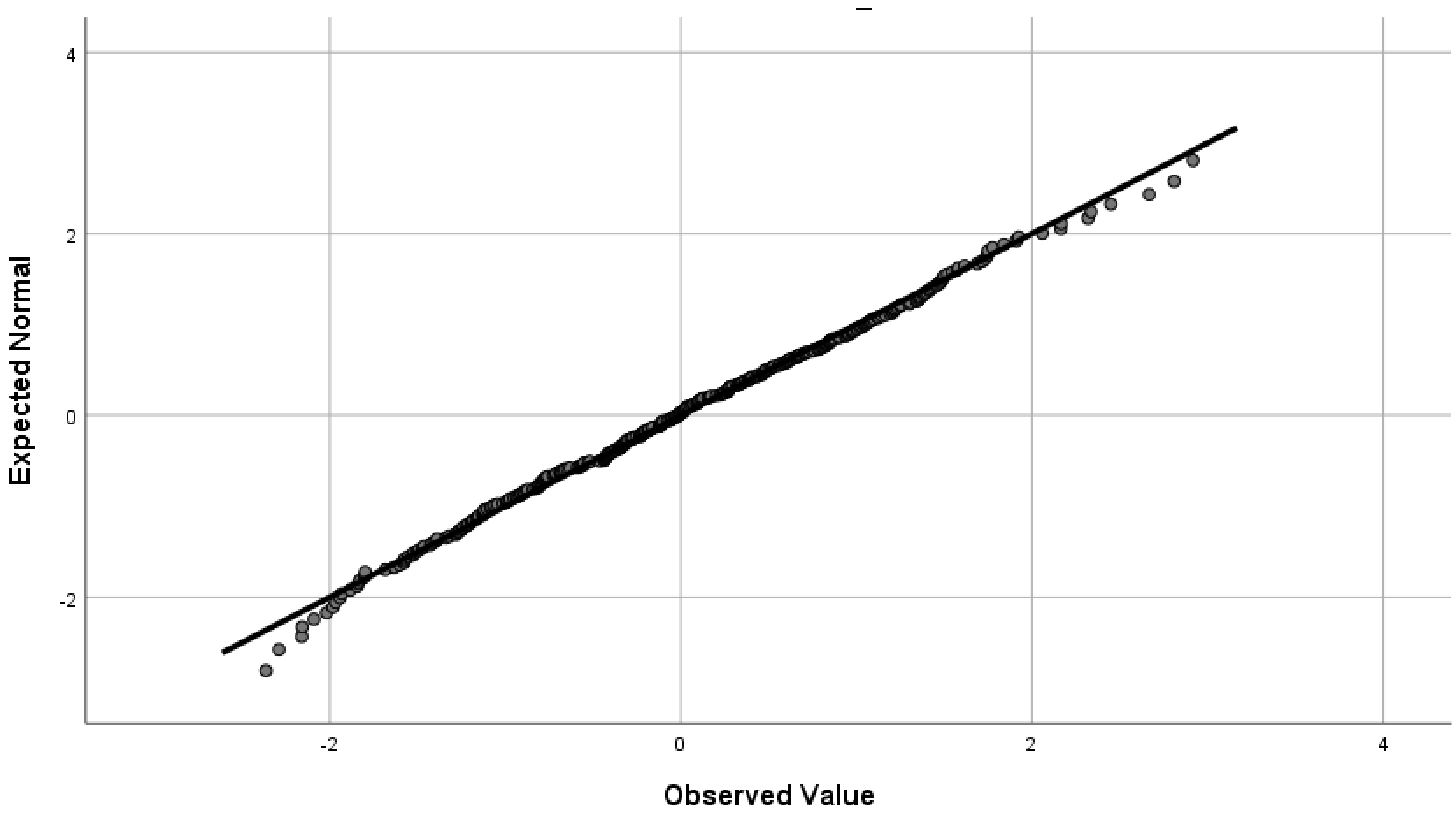

Figure S3

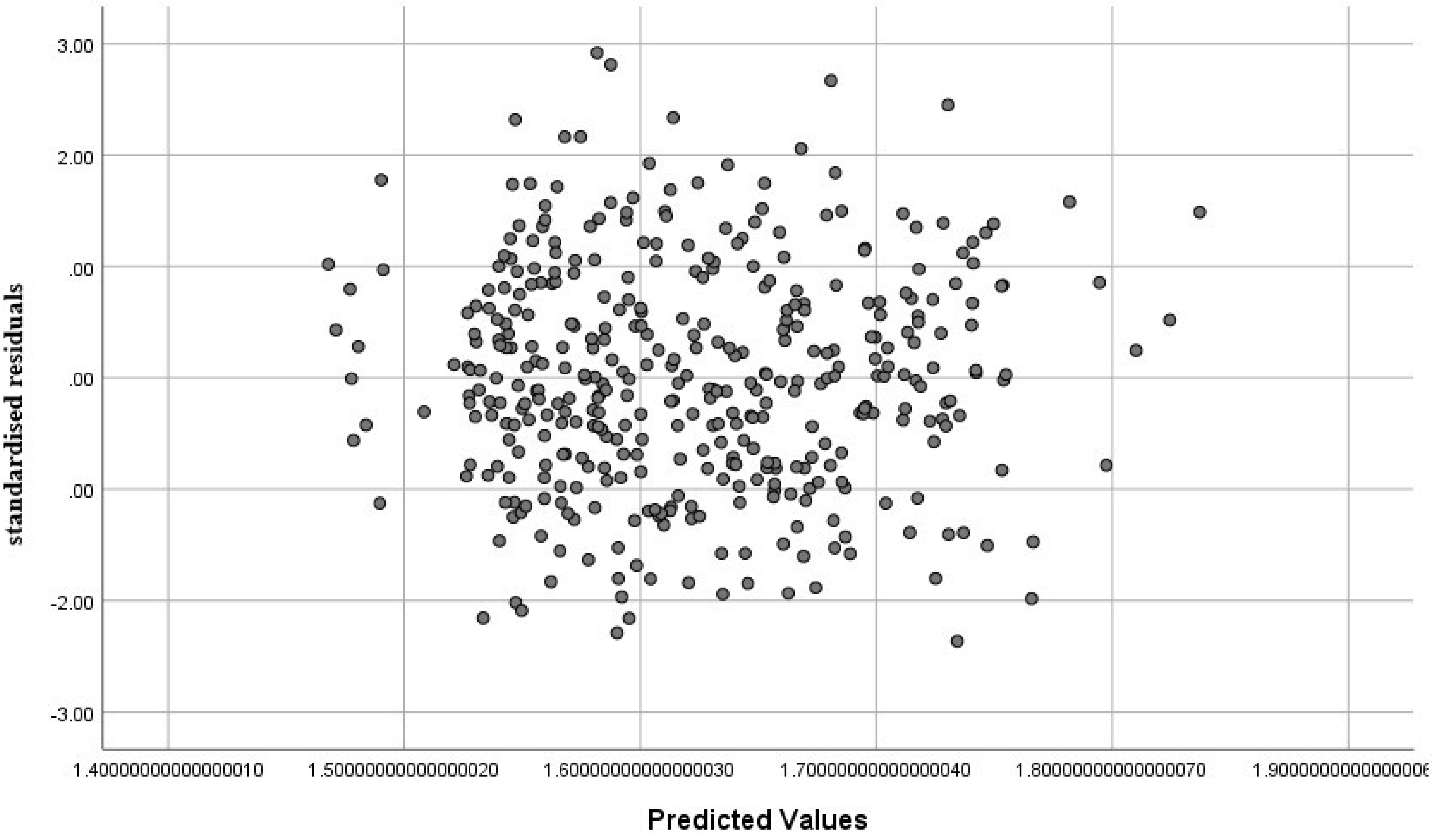

3.00
2.00
.00
.00
.00
-2.00
-3.00
standardised residuals
1.4000000000000010
1.5000000000000020
1.6000000000000030
1.7000000000000040
1.8000000000000070
Predicted Values

Figure S4

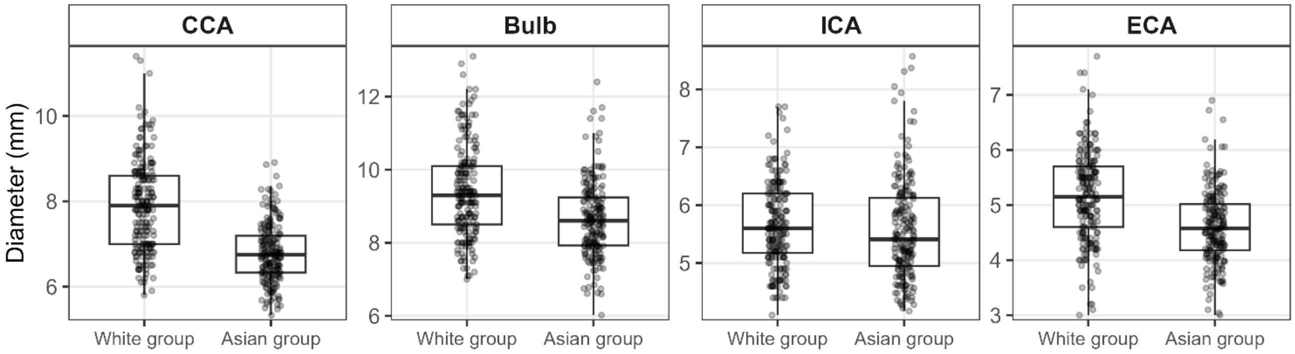

CCA
Bulb
ICA
ECA
Diameter (mm)
10
8
6
12
10
8
6
8
7
6
5
7
6
5
4
3
White group
Asian group

.

Figure S5

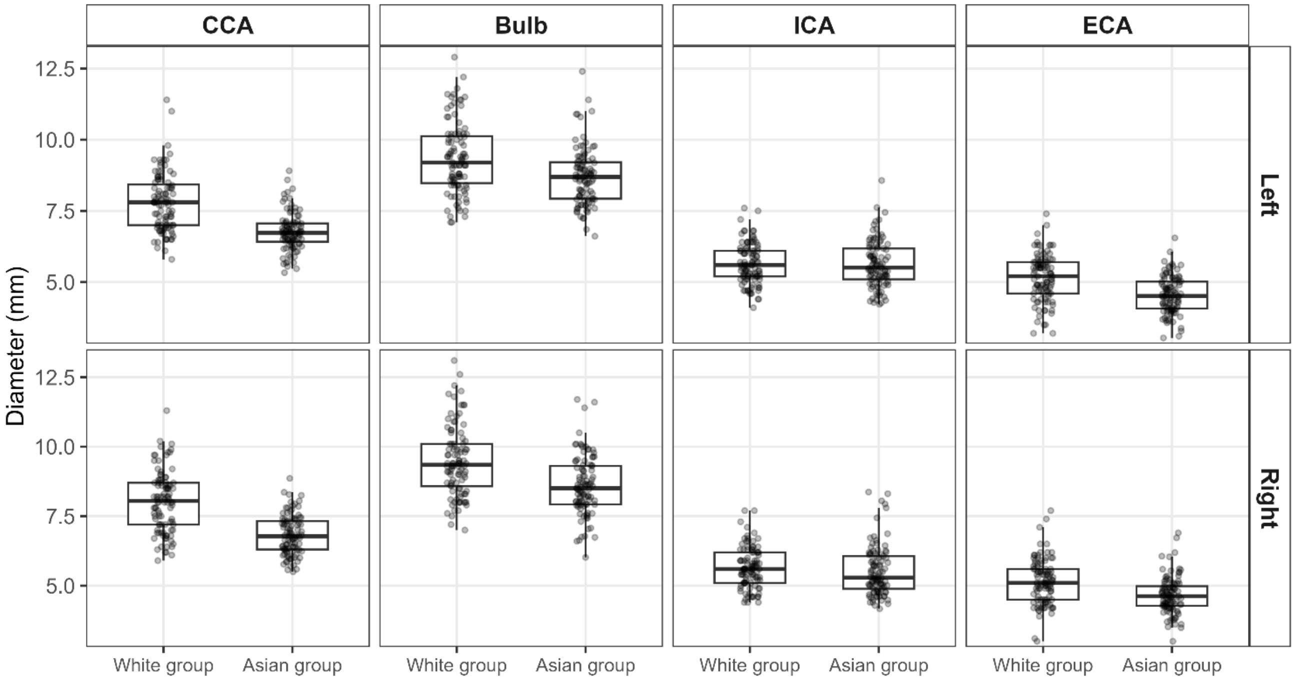
CCA
Bulb
ICA
ECA
Left
Right
Diameter (mm)
12.5
10.0
7.5
5.0
White group
Asian group

Figure Legends

Figure S1. Distribution of standardized residuals from the linear mixed-effects model with Carotid bulb (CB)/internal carotid artery (ICA) as outcome.

Figure S2. Q–Q plot of standardized residuals from the linear mixed-effects model with Carotid bulb (CB)/internal carotid artery (ICA) as outcome.

Figure S3. Standardized residuals plotted against fitted values for the primary mixed-effects model with Carotid bulb (CB)/internal carotid artery (ICA) as outcome.

Figure S4. Unadjusted mean ± standard deviation of absolute carotid bulb and internal carotid artery diameters stratified by Ethnicity. For each participant, left and right measurements were averaged to obtain a single representative value per vessel, thereby reducing within-subject correlation. Values are presented for descriptive and exploratory purposes only.

Figure S5. Unadjusted mean ± standard deviation of absolute carotid bulb and internal carotid artery diameters stratified by Ethnicity and side (left and right analyzed separately). These data are provided for descriptive completeness and to illustrate side-specific vessel dimensions; no inferential statistical testing was performed.
